# TWIICE One powered exoskeleton: effect of design improvements on usability in daily life as measured by the performance in the CYBATHLON race

**DOI:** 10.1186/s12984-022-01028-0

**Published:** 2022-06-27

**Authors:** Tristan Vouga, Jemina Fasola, Romain Baud, Ali Reza Manzoori, Julien Pache, Mohamed Bouri

**Affiliations:** 1 TWIICE SA, Lausanne, Switzerland; 2 Sonceboz SA, Sonceboz-Sombeval, Switzerland; 3 Biorobotics Laboratory (BioRob), EPFL, Lausanne, Switzerland; 4 Translational Neural Engineering Laboratory (TNE), EPFL, Campus Biotech, Geneva, Switzerland

**Keywords:** Powered exoskeleton, Wearable robotics, Powered gait orthosis, Spinal cord injury, Overground walking, Gait, Exoskeleton training

## Abstract

**Background:**

Spinal cord injury leading to paraplegia affects the mobility and physiological well-being of one in a thousand people. Powered exoskeletons can temporarily restore the ability to walk. Their relevance in daily life is still limited because of low performance beyond ground that is even. CYBATHLON is an international competition promoting improvements in assistive technology. In this article, we present the latest design and results of testing of TWIICE One version 2018, one of the competing devices in the 2020 race.

**Methods:**

A person with a motor-complete spinal cord injury at thoracic level T10 participated as race pilot. Training ahead of the race took place over one week at a rate of 2 h per day. The time to perform each of the seven tasks of the competition was recorded together with the number of repetitions. Performance is compared over the training period and against the 2016 race results.

**Results:**

Progression was observed in all tasks and accounted for by both user training and technology improvements. Final competition rank was second out of seven participating teams, with a record time of 4′40". This represents an average improvement of 40% with respect to comparable obstacles of the 2016 race, explaining the two ranks of improvement since then.

**Conclusion:**

These results help understand which features had a positive impact on the real-life performance of the device. Understanding how design affects performance is key information to create devices that really improve the life of people living with paraplegia.

## Background

Restoring functional mobility in people with motor-complete spinal cord injury (SCI) is one of the targeted applications of powered lower limb exoskeletons [1, 2] in which significant progress has been made over the past decade. Serious efforts in the development of such devices at least dates back to the late twentieth century [3], and advances in robotics-related technologies have facilitated these developments. Significant improvements have been achieved since the early proof-of-concept studies showing the feasibility of powered orthoses to enable SCI patients’ ambulation [4], and several commercialized or market-ready solutions already exist today [5–11]. Studies also have clearly shown the desirability of these devices for people with SCI [12, 13].

These devices can be used by a wide range of subjects [14] and offer numerous advantages compared to passive devices and wheelchairs, including improved mobility [15], enabling locomotion over different types of terrain [16], and increased motivation for physical activity [13, 17], which in turn lead to physical [18, 19], emotional/mental and social benefits [20–22]. Improved mobility, social functionality and the ability to carry out activities of daily living (ADLs) are important factors for improved quality of life in people with SCI [23, 24]. Various other potential benefits and advantages of using such devices also have been widely reported in the literature [25].

However, despite the positive points, these devices are not yet recognized as suitable for completely independent everyday use. They have been proved useful mostly in clinical and controlled settings [26–28], despite preliminary evidence for the feasibility of their use in realistic settings [25, 29]. Factors such as safety concerns, the need for supervision and/or external supporting structures [26], reliance on crutches, slow or robot-like walking [12], high prices [30] and their limited availability [27] make the widespread adoption of such devices for everyday use impractical. Moreover, the risk management and regulatory systems for these devices are not mature yet and require better understanding of the various aspects related to their use [31]. These issues highlight the necessity of further development of the exoskeletons [26] and more studies on their usage in realistic settings [31]. Improvements in controllers and trajectories can also further improve the functional outcomes [32].

One of the major drivers for the development and adaptation of assistive technologies to real-world scenarios in the recent years has been CYBATHLON, a competition for people with physical disabilities using advanced assistive technologies. One of its disciplines is the powered exoskeletons race [37] which consists in a set of tasks representative of the ADLs. The exoskeleton users (referred to as “pilots”) must be motor complete thoracic or lumbar SCI persons, with paraplegia and a complete loss of motor function in the lower limbs (AIS A or B) while having sufficient voluntary control and strength to either hold crutches, stabilize the trunk or both. The metrics of success are firstly versatility (i.e., solving as many tasks as possible), and then agility (i.e., accomplishing the tasks as quickly as possible, and within a given time limit). The rules of the race also attempt to mimic the conditions of realistic ADLs, for example:

- All components (e.g., batteries, control units, tools, spare parts, etc.) used with the device must be carried only by the pilots from the start to the end of the race.
- Only the pilots are allowed to maintain or replace components of their device during the race.
- Communication between the device and any third-party stationary site for the purposes of controlling or tuning is not allowed during the race.

There are also criteria on the design of the exoskeletons, such as a weight limit of 85 kg for the device, prohibition of load transfer to the ground via wheels or rolling contact, and prohibition of using combustion engines for actuation [38]. The tasks and criteria of this race have guided the development of several lower-limb exoskeletons, as previously reported in the literature [39–41]. One of these exoskeletons is TWIICE One version 2018, which empowered the silver medalist in the CYBATHLON 2020.

The development of the first version of TWIICE One started in 2015, with the aim of creating a modular, compact, and lightweight lower-limb exoskeleton for people with paraplegia. With the principle of simplicity in mind, only the hip and knee joints were selected to be actuated. The ankle has been locked, but the lack of this degree of freedom (DOF) was compensated with a curved sole, one of the unique features of TWIICE One. The development of the first version (TWIICE One version 2016) which was finalized in 2016 [42], has been driven by our participation in the first CYBATHLON the same year. Based on observations during the training sessions and the CYBATHLON Experience events, and the pilot feedback, the software was improved gradually, and design notes were collected. This led to designing a new device, TWIICE One version 2018, with improved mechanical structure, actuators, and electronics, which is described in this paper.

In the rest of this paper, first the mechanical design and control of the 2018 version will be explained, and the major changes as compared to the 2016 version will be highlighted. Then, the pilot of the device and the tasks of CYBATHLON will be described in the Methods section. The achieved functional outcomes, including overall performance and the obtained results in the CYBATHLON 2020 will be presented, followed by a discussion of how different changes in the design affected the performance. Finally, the key points will be revisited and summarized in the “Conclusion” section.

## Methods

### Mechanical design

#### Overview

**Fig. 1:**
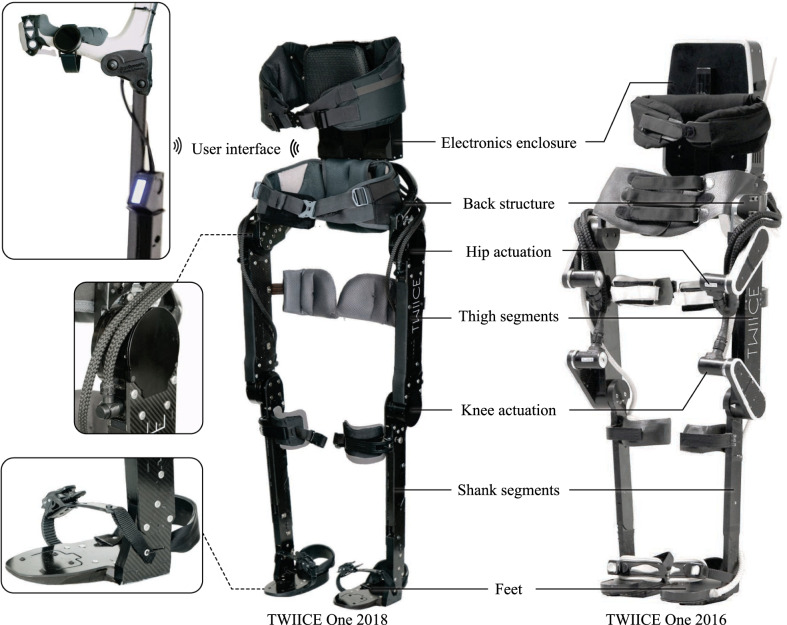
TWIICE One 2018 overview and a direct comparison with the 2016 version. The inlays, from top to bottom, show the user interface comprising a set of buttons as well as a trigger on the right crutch handle, a smartwatch and an LCD display as visual feedback, a zoom on the custom motors, and a zoom on the foot design

TWIICE One is a powered hip-knee-ankle–foot orthosis comprising four actuated joints in total and no passive joint. The structure maintains the user’s lower limbs from torso to foot, using five attachment points: a thoracic belt, a waist belt, thigh and tibial cuffs and foot straps (Fig. 1). The active joints mobilize the hips and knees in the sagittal plane, so that gait motion is made possible to users with a motor-complete spinal cord injury. Batteries, as well as controls and power electronics are placed in the back of the user in a robust enclosure rigidly mounted on the device’s back structure. Adjustment possibilities include length of the tibial and thigh segments, width of the pelvic structure and ab-/adduction angle. The thoracic belt is also height-adjustable to accommodate each user's level of trunk control. Power and signal distribution to the actuators is done by multipolar cables protected with mechanical and electromagnetic shielding. Power and digital lines are combined in the same cable assembly using Fischer Connectors’ Alulite connectors [43]. Peripherals include forearm crutches with user input device and an off-the-shelf smartwatch. Construction materials are primarily long carbon fiber reinforced epoxy built in sandwich arrangement for the thigh and tibia segments and aluminum for the pelvic structure. User attachment points are made of technical breathable fabric and lightweight foam. In total, the device weighs 16 kg including the batteries.

#### Design approach

The architecture and design philosophy of TWIICE One exoskeletons is revolving around simplicity, with the underlying intention to achieve more robustness and lower weight. From the number of actuators to the manufacturing methods, a minimalistic approach was adopted in design decisions. This strategy led to lower implementation costs, reliable operation over the years and light weight. Over 20′000 steps were taken with the prototype during development tests and no hardware failure occurred. Hardware maintenance remained low with no significant part replacement apart from a foot sole and one fatigue failure of the trigger used for user input.

#### Actuation

The four actuators are identical in design. They consist of a brushless DC motor and a cycloidal gear with ratio 1:51 custom made by the company Sonceboz SA [44]. Its rated torque is 100 Nm, limited by the mechanical capacity of the gear. The motor’s custom design had the following key design objectives: high torque density, low back driving torque, and safe mechanical failure mode. The resulting key design performance metrics are detailed in Table 1. Other features include: a relative position encoder on the motor’s rotor, a thermistor on the stator windings, an absolute position encoder measuring the joint angle, signal conditioning and sampling performed locally with digital communication bus to the embedded computer in the back, and mechanical stops at anthropomorphic joint limits in flexion and extension. The failure mode of cycloidal gears is blocking by design. When undergoing overstress, the teeth which normally have enough clearance start interfering and block the input disks before tooth stripping. This inherently safe design means a lower safety factor can be used for the gear without compromising safety of the system. In turn, this yields a more compact and lighter system. The total weight per actuator is 1.63 kg, with an external diameter of 85 mm and axial length of 55 mm.

**Table 1:** Technical specifications, typical preparation time, walking speed and adjustability of the exoskeleton TWIICE One 2018. The information is layout the same as Schrade et al.'s paper [39] for easier comparison

Specifications	Unit	
Weight	kg	16
Intermittent peak torque	Nm	100
Max. joint velocity	rpm	40
Battery life	h	3
Typical preparation time	s	60
Typical don/doff time	s	129/75
Typical walking speed	m/s	0.32
Hip width	m	0.340–0.400
Thigh length	m	0.385–0.455
Shank length + ankle height	m	0.431–0.533

#### Feet

Simplicity and light weight were achieved partly through reduction of the number of degrees of freedom. To compensate for the lack of mobility at the ankle, a special sole shape was designed to allow for easy lateral weight shift and to roll over the floor during stance phase. To manage rough terrain and navigating narrow spaces, the foot design was further improved with respect to TWIICE One version 2016. Compliance was added in the soles to allow the fore part of the foot to flex with respect to the main base at the metatarsal level. The overall length and height of the sole were also reduced to facilitate interaction with the ground and irregularities.

### Control

#### User interface

TWIICE One is controlled by the pilot via two main input devices: (i) buttons on the crutch handle and (ii) an Android smartphone or smartwatch installed with an interface application (Fig. 1, top left inlay). The set of buttons on the crutch include three push buttons and a trigger button. They are pressed respectively with the thumb and the index finger, while the hand holds the crutch handle. The “up” and “down” buttons are mostly used to navigate between the modes while the round button allows to exit them. The trigger is used to initiate actions (e.g., entering a mode or taking a step). The smartwatch is used to send higher-level commands that are not used during walking, such as (de)activating the motors or shutting down the exoskeleton. Besides this functionality, the smartwatch principally serves as a visual feedback device, displaying the selected/active mode to the user, and the battery state. To mitigate the risk of connection loss between the smartwatch and the exoskeleton, a wired LCD display was integrated into the crutch. The name of the current mode is displayed on this extra screen, which is wired to the embedded computer of the exoskeleton.

#### Controller behavior

As described in [41], the control of TWIICE One is based on several pre-recorded trajectories. Pre-defined trajectories are predictable and can be learnt by the user. This facilitates the contribution of the trunk and the upper limbs to maintain balance. However, there is no automatic adaptation to the terrain. Increasing the diversity of the gait trajectories addresses more situations but results in a longer time to switch modes and is also more user error prone.

A new feature has been added to be able to trigger a mode-specific secondary action with the “up” and “down” buttons. This feature is used by the following modes:

- Periodic gait mode: The “up” and “down” buttons switch smoothly from one gait trajectory variant to another (e.g., different step sizes and stair heights). This avoids the need for numerous gait modes.
- Tilted path mode: The “up” and “down” buttons offset vertically the foot locus of one side with respect to the other. This is useful for walking through a terrain inclined laterally, such as the tilted path obstacle of the CYBATHLON.
- Rough terrain mode: The “up” button triggers a higher and longer step to walk over a small obstacle (~ 10 cm height). The “down” button triggers a higher step to walk on a small obstacle (~ 10 cm height). These special steps have been designed specifically for the “rough terrain” obstacle of CYBATHLON 2020.

For the CYBATHLON 2020, 9 modes were available to the TWIICE pilot to overcome the six obstacles (sofa sitting, normal gait, fast gait, rough terrain, stairs ascent, stairs descent, tilted path, slope ascent, slope descent).

#### Gait trajectories design

For TWIICE One version 2016 [42], the design of the gait trajectories was done from a list of joint angles associated to the strides in the gait cycle. A MATLAB script (Mathworks Inc., Natick, MA, USA) interpolates them over the full gait cycle and displays the corresponding joint angles, joint velocities and the resulting foot locus obtained by forward kinematics. This allows manual tuning. It eventually generates C-code to be compiled with the exoskeleton controller. The entire process typically required about 15 min for a moderate change in the gait trajectory.

To make this procedure quicker, a dedicated application was developed with a graphical user interface, which allows designing the foot locus trajectory while displaying the joint-level outcome in real-time. The application then generates foot locus trajectory files that can be directly used by the exoskeleton’s controller. Inverse kinematics are solved on-board. The trajectories can be updated at runtime, which allows testing different trajectories with minimal downtime (< 2 min for a moderate change). This enables fast iterative adaptation of the trajectories for different tasks.

### Pilot

One T10 sensorimotor complete spinal cord injured (AIS A) pilot took part to the development, test, and validation of the usability of the exoskeleton. The test pilot was the same athlete as in the CYBATHLON 2016 and has therefore regularly trained (i.e., sit-to-stand transition, in- and outdoor walking and stairs climbing) with the exoskeleton for four years. She has sufficient voluntary control of shoulders, arms, and neck to keep her upper body upright and use the crutches to balance, but needs assistance, provided by an upper belt, to maintain her trunk upright. The pilot does not have any restriction in the range of motion of the hips and knees but has some ankle stiffness that decreases progressively when loaded regularly. Detailed information of the pilot is found in Table 2.

**Table 2:** Detailed information on the test pilot

Specifications	Unit	Pilot
Age	Years	48
Weight	kg	45
Height	m	1.58
Gender		Female
Years post injury	Years	13
Level of injury		T10-T11
AIS classification		A
Self-reported clinical syndrome		Neuropathic pain, low spasms
Previous experiences with exoskeletons	TWIICE One 2016	CYBATHLON 2016 CYBATHLON experience 2017
	TWIICE One 2018	CYBATHLON experience 2018 and 2019

The testing and development were performed in agreement with the guidelines for technical assistance devices/medical devices from Swissethics [45]. The test user gave informed consent to participate in the tests and the training.

### The CYBATHLON 2020 as a benchmark for performance evaluation

The CYBATHLON 2020 race is used as a benchmark to describe the performance of the exoskeleton. The race included (1) a “sit & stand” task requiring the pilot to sit and stand up from a 460 mm high sofa and walk to a table to stack cups, (2) a “slalom” task challenging the pilot to navigate around furniture, (3) a “rough terrain” task that required accurate control of foot positioning, (4) a “stairs” task that tested the exoskeleton ability to climb up and down stairs, (5) a “tilted path” task that included a ramp inclined perpendicularly to the walking direction, and 6) a “ramp & door” task that required the pilot to ascend a 20° slope and descend a 15° degree slope and navigate through a door while walking on flat ground.

The number of training sessions, their durations, and the best time for each obstacle are reported to show the pilot’s progress in controlling the device, as well as the effects of the new sole design and a different walking strategy for the rough terrain obstacle. The sessions duration includes the exoskeleton preparation, donning and doffing of the device.

The number of repetitions per obstacle all training sessions combined is given to demonstrate which obstacles were the most challenging and required more practice.

The best time per obstacle for the three heats of the CYBATHLON 2020 race are given as performance indicator. Although the obstacles of the CYBATHLON 2016 were slightly different, their times are given as a baseline to evaluate the combined improvement of the device design and the pilot’s skills.

### Usability evaluation

The usability of the exoskeleton is evaluated with the preparation time, donning and doffing time, as well as the walking speed. The preparation time includes the time needed to connect the battery, start the device and check that the interfaces are open for the transfer. Donning and doffing comprise the exoskeleton/wheelchair transfer, and the fastening/detachment of the interfaces by the pilot without assistance, respectively. The average and standard deviation of two timed donning and doffing sessions are reported [46]. The typical and the fastest walking speed has been evaluated with a 10 m walk test (10MWT) using the normal gait and the fast gait modes. Pilot satisfaction has not been evaluated in a systematic way.

## Results

### Exoskeleton design

To assess the actuator design, we evaluate the torque delivered by the motors during several activities. This indication is relevant to validate the design choices that governed the actuator development. Both the motor and the reducer were designed based on specifications which were stemming from estimates using extrapolated data from previous designs and from theoretical rigid body models. These approaches present important limitations when attempting to estimate the losses in the reducer and the divergences between model kinematic parameters and real-life motion of the user and system in space. Measuring the torque of the actuator during different Cybathlon activities sheds light on the actual torque requirements for use in daily life. Kinematic measurements are made using the joints proprioception sensors mounted on the rotor assembly. Torque measurements are calculated based on the current measurement of the windings and the motor’s measured mechanical constant.

**Fig. 2:**
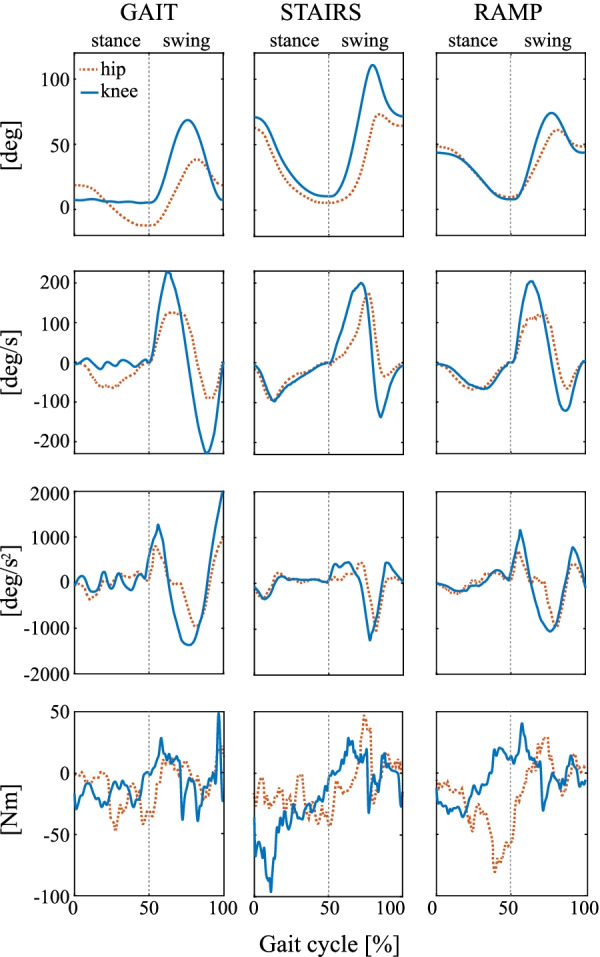
Joint kinematics of the hip (dashed line) and the knee (solid line), their angular velocities and accelerations, and their torque for normal gait, stairs and ramp ascent represented as a function of stride. The vertical line represents a pause in the gait cycle between the stance and swing phases, corresponding to the time needed by the pilot to move the crutches forward

Figure 2 compares the kinematic parameters of the hip and knee during the gait cycle of three activities: overground level walking, stair ascent and ramp ascent. Kinematic parameters are given in position, velocity, and acceleration for each activity and joint, in both stance and swing phases. The torque delivered by the motor at each joint is shown in comparison below the kinematic parameters.

The highest torque peak occurs unsurprisingly during stance of the stair ascent during the knee joint extension. With a magnitude of 100 Nm, this parameter should be the main factor for the actuator’s reducer and motor. Another important sustained torque peak occurs in flexion of the hip during swing of the leg in stair ascent. Because of their non-negligible duration, these two events should be considered for the further design and development of exoskeleton actuators. Other important landmarks of the torque profiles are the knee flexion torque peak at the end of the level gait swing. Provoked by the sudden deceleration of the knee joint, this peak also reaches 50 Nm but for a much shorter duration. Finally, the hip extension during ramp ascent swing to lift the body up is significant in duration. This should be watched closely when specifying the motor rated torque as limited by thermal effects.

### CYBATHLON performance

**Fig. 3:**
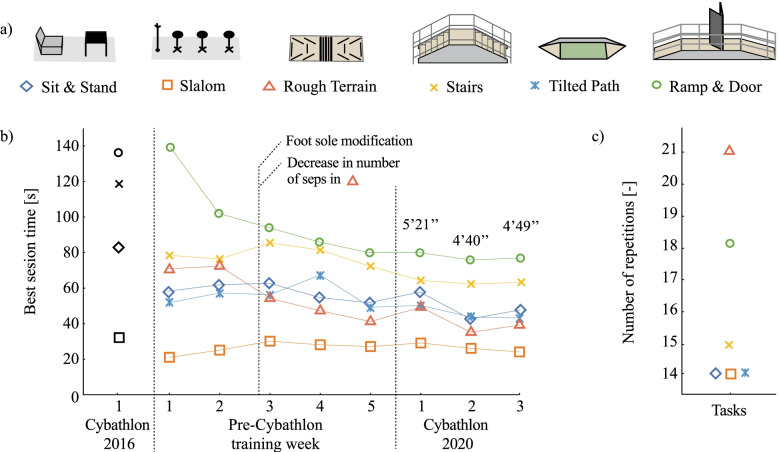
Performance on the CYBATHLON obstacles. **a** The six CYBATHLON 2020 obstacles, matching the race order from left to right. **b** Best times per obstacle. The whole race times are given for the three heats of the 2020 competition. **c** Number of repetitions per obstacle during the training week before the CYBATHLON 2020 competition

As the pilot was experienced and had limited availability ahead of the competition, five intensive training sessions of two hours each were scheduled one week before the CYBATHLON 2020 competition. The obstacles were mostly practiced in sequence and in the same order as in the competition to account for the exertion factor throughout the race. The best times per obstacle from the CYBATHLON 2016 and CYBATHLON 2020 training sessions, as well as all three CYBATHLON 2020 heats are shown in Fig. 3. We observe that the “sit & stand” task was up to 37 s faster than in 2016 although an additional standing agility task was added. The “slalom” task increased in difficulty with bulkier objects to circumvent and narrower spaces. However, time to complete this task decreased by 13 s between 2016 and the first training session. To increase gait stability and preserve the pilot from fatigue, it was decided to reduce the pace after the first training. The “rough terrain”, not performed in 2016, was passed with a best time of 36 s. This time was drastically reduced in session number three with a modified foot design and a new stepping strategy. With a shorter foot sole and an additional passive elastic joint in the toes, longer steps with a high foot clearance could be taken over the ground obstacles. Moreover, the sole flexibility allowed to partially step on the obstacles without affecting the whole-body balance. The “rough terrain” was the most trained task, along with the “ramp and door” task. The “stairs” task was the same as in 2016, and the record was broken by 55 s due to pilot skills improvement. Without hip abduction/adduction or pronation/supination in the ankle, the “tilted path” could be traversed in 42 s using an asymmetric gait pattern. Finally, the “ramp & door” task was crossed 58 s faster than in 2016. The flexible toe of the sole allowed for a better posture and grip on the ground, avoiding a yaw rotation of the whole body when in single stance. The record time of 4′40’’ for the entire race was achieved during the second round of the competition. This represents an average of 40% improvement with respect to comparable obstacles of the 2016 race.

### Usability

The device preparation time was evaluated to 60 s. The pilot can transfer inside the exoskeleton in 129 ± 12.7 s (donning time) and can doff of the device in 74.5 ± 0.7 s. The pilot has a walking at 0.32 m/s with the normal gait mode, which has a shorter step length than the fast gait allowing to negotiate turns with ease. This mode is mostly used in narrow spaces or in a crowded environment. The pilot reaches a maximum speed of 0.40 m/s which is 0.04 m/s faster than in 2016 and above the mean of four studies with other devices including a total of 53 AIS A patients (mean = 0.35 ms^−1^, SD = 0.12) [16, 20, 47, 48]. At such speed, the gait cadence is 1.1 step/s. Fast gait mode is mostly used for walking in a straight line.

## Discussion

TWIICE One 2018 is an evolution of TWIICE One 2016 and keeps its most important design points, which are the composite mechanical structure and the four actuated DoFs. In addition, the curved sole of the foot compensates for the lack of a flexible ankle, and allows turning easily, thanks to the small area of contact with the floor. The control strategy also remains position-control, with operation modes to set the joint trajectories. Every step or other action is triggered by the fingers of the pilot, using the four buttons on the right crutch handle.

This simple design makes TWIICE One lightweight and predictable. However, due to the lack of actuation at the ankle and the pre-defined trajectories, walking across unstructured terrain or an obstacle not handled by a specific control mode is difficult for the pilot. The contribution from the arms and the crutches is then increased, to ensure the stability.

TWIICE One 2018 brings an improved mechanical design to increase the joints torque and the compactness. This was possible thanks to the custom integrated actuator, including the motor, gears, and sensory electronics. Compliance was also added to the curved sole.

The embedded controller was also extended with additional operating modes to address two new CYBATHLON obstacles of: the “tilted path” (different in 2016) and the “rough terrain”. The gait trajectories were optimized by trial-and-error thanks to a desktop application allowing quick iterations. Manual control of the exoskeleton by the pilot using the buttons on the crutch also turned out to be a good strategy and was maintained in the new version. This method of control, in combination with predefined trajectories, has made the interaction between the pilot and the exoskeleton simple, thanks to the predictability of its behavior. This, however, comes at the cost of inflexibility in the face of uncertainties in the environment. This problem could be mitigated by providing several locomotion modes, each with a trajectory designed for a specific type of terrain.

The main outcome is a better time to go through the CYBATHLON obstacles, compared to the 2016 edition.

Usability was assessed with the preparation time, donning and doffing time, and the walking speed. The user satisfaction was not evaluated in a systematic way as only one pilot was enrolled. However, the design was user-centered as the pilot feedback was the main source of inspiration. It can be noted that the gait speed did not increase significantly. This is due to the gait trajectories being triggered step by step, requiring a short pause in the middle of the stride. Indeed, all the joints are fully stopped at the end of each step, so time is lost to decelerate, wait for the user to move the crutches, and then trigger the step, and accelerate again.

The main limitation of these results is that the performance of TWIICE One was assessed with a single pilot only. Chosen for her athletic condition, her performance is not necessarily representative of most people affected by paraplegia. The performance indices exposed here should then be considered as the maximum achievable, not the typical ones.

In the future, the controller of TWIICE One will be improved to support a continuous gait, to increase the ambulation velocity. To allow walking in unstructured terrain, adding more gait trajectories is not sustainable because of the increased time to select them, and the increased risk of error. Terrain-awareness and dynamically-computed gait could be implemented if extra sensing is added to TWIICE One, using a LIDAR distance sensor.

## Conclusions

In this paper, we presented the evolution of TWIICE One, a powered hip-knee-ankle–foot orthosis for persons with SCI and reported the achieved functional outcomes in terms of performance in the CYBATHLON competition. The outcome of two years of development, TWIICE One 2018 is actuated at the hip and knee levels and benefits from a curved sole similar to its 2016 predecessor. However, the design has been improved with structural flexibility of the feet, adjustability of segment lengths and more powerful actuators. The minimalistic design approach proved to be a successful trade-off, resulting in a low-cost implementation, compactness, robustness against failures and minimal maintenance requirements thanks to fewer components.

In addition to the mechanical modification, some of the locomotion modes have been improved with parameters that are tunable by the pilot during operation. The accelerated optimization of the predefined trajectories, thanks to an efficient design interface, was found to have remarkable potential in improving the overall speed and ease of use of the exoskeleton.

The outcome of these improvements was reflected in a notably faster performance in the CYBATHLON 2020 and winning the second place, with a reduction of up to 48% in the time required for some of the tasks compared to 2016. Despite the modifications in hardware and software, our pilot was not only able to easily adapt to the changes, but also improved her performance significantly with minimal training. This was possible thanks in part to the pilot’s previous experience with the exoskeleton, even after a long hiatus imposed by the sanitary restrictions due to the COVID-19 pandemic. This highlights the importance of initial training for exoskeleton users and suggests that the acquired skills can persist over an extended period.

## Data Availability

All data is presented in the article.

## Abbreviations

SCI: Spinal cord injury
ADL: Activities of daily living
DOFs: Degrees of freedom
